# HIV, co-morbidity and ageing

**DOI:** 10.7448/IAS.15.6.18073

**Published:** 2012-11-11

**Authors:** P Reiss

**Affiliations:** Academic Medical Center, University of Amsterdam, Division of Infectious Diseases and Department of Global Health, Amsterdam, The Netherlands

## Abstract

As treatment for HIV infection needs to be used continuously and lifelong, issues concerning long-term outcomes, including those involving tolerability and safety of treatment, are gaining increasing importance. Although current combination antiretroviral therapy (cART) regimens are generally better tolerated than those in the early days of cART, treatment toxicity remains an important cause for discontinuation of (components) of treatment. Moreover, several of the potential toxicities of cART (including cardiovascular, metabolic, renal and bone toxicity) overlap with known ageing-associated co-morbidities. Given that our patient population with HIV is increasingly getting older as a result of the success of cART in reducing traditional HIV-associated morbidity and mortality, these co-morbidities are increasingly being seen and importantly influence patient management. Moreover, persons with HIV, in spite of having suppressed viraemia on cART seem to be at increased risk of the premature development of age-associated non-communicable co-morbidities, including cardiovascular, chronic kidney, liver and pulmonary disease, diabetes mellitus, osteoporosis, non-AIDS associated malignancies, and neurocognitive impairment. It has therefore been hypothesised that such individuals, despite effective cART, may be prone to accelerated ageing. The underlying pathogenesis is likely to be multifactorial and, apart from include sustained immune activation, both systemically and within the central nervous system. The presentation will review the current state of knowledge and investigation in this area.

